# Effect of *Withania somnifera* Extracts on Some Selective Biochemical, Hematological, and Immunological Parameters in Guinea Pigs Experimental Infected with *E. coli*

**DOI:** 10.1155/2013/153427

**Published:** 2013-03-31

**Authors:** Mohamed El-Sayed El-Boshy, Osama Mohamed Abdalla, Angy Risha, Fatma Moustafa

**Affiliations:** ^1^Department of Clinical Pathology, Faculty of Veterinary Medicine, Mansoura University, Mansoura, Egypt; ^2^Laboratory Medicine Department, Faculty of Applied Medical Science, Umm Al-Qura University, PB 7296, Makkah 21955, Saudi Arabia; ^3^Department of Clinical Pathology, Faculty of Veterinary Medicine, Suez Canal University, Ismalia, Egypt

## Abstract

Fifty 1-2-month-old Guinea pigs were divided into 5 equal groups, 10 each. Control (Gp1) did receive neither viable bacteria nor treatment. Each animal from the other groups (Gp2–5) was challenged with (1-2 × 10^8^) viable *E. coli* in 200 **μ**L normal saline (0.9%) through IP route. GP2 infected group was treated with 200 **μ**L saline IP and kept as positive control group. Gp3-4 are infected and treated with *Withania somnifera* (ethanol root extract) with doses 50 and 100 mg/kg. BW, respectively. Gp5 infected treated group was treated with cefoperazone antibiotic at dose 35 mg/Kg BW. The treatment by drug or the extracted medicinal plant was started 72 h after infection for 7 successive days. Serum and whole blood sample were collected from all groups 14 days after treatment to evaluate some hematological and biochemical changes as well as immunomodulatory cytokine tumor necrosis factor-alpha (TNF-**α**). Oral treatment of the plant extract caused significant benefit results in infected Guinea pig appeared in the correction of some hematological and biochemical parameters also try to suppressed inflammatory cytokine response represent in TNF-**α**. It could be concluded that *W. somnifera* extract has potent antibacterial activity, and this appears in the correction with hematological, biochemical, and immunological results.

## 1. Introduction 

In the last few years, there has been an exponential growth in the field of herbal medicine, and these drugs are gaining popularity both in developing and developed countries because of their natural origin and less side effects. Many traditional medicines in use are derived from medicinal plants, minerals, and organic matter [1]. Research work on medicinal plants and exchange of information obtained will go a long way in scientific exploration of medicinal plants for the benefit of man and is likely to decrease dependence on imported drugs [2]. The chemical diversity, structural complexity, lack of substantial toxic effects, and broad spectrum of antiviral and antibacterial activity of natural products make them ideal candidates for new therapeutics [3].

*Withania somnifera* is an important medicinal plant, a small, woody shrub 60–200 cm high, in the Solanaceae family, which is described under many commen names such as Ginseng and Ashwagandha. It can be found growing in Africa, the Mediterranean, and India. The roots are the main portions of the plant used therapeutically [4, 5]. Withanolides are the major active constituents of *Withania somnifera *that are isolated from its root and leaves. Recently, the plant was investigated to be effective against treatment of some bacterial infection and tested for serious antibacterial properties [6–8]. Besides its, uses as antibacterial product several reports have demonstrated immunomodulator and tumor activity of this extract [9–11].

Cefoperazone is a commonly used broad spectrum semisynthetic third-generation cephalosporin with a potent bactericidal activity against a wide range of Gram-positive and Gram-negative bacteria [12]. Moreover, differs from other beta-lactams in the extent to which it is excreted in the bile and the stability of its elimination pharmacokinetics in the presence of renal impairment [13].

The objective of the present work is to evaluate antibacterial activity of *Withania somnifera *as well as some biochemical, hematological, and immunological parameters in Guinea pigs experimental infected with pathogenic *E. coli.*

## 2. Material and Methods

### 2.1. Preparation of Extracts from Root and Leaves of *W. somnifera*

*Withania somnifera* root and leaves part were collected from Faculty of Agriculture, Mansoura University. The samples were taxonomically identified at the Botany Department, Faculty of Science, Mansoura University. Plant materials were dried in the dark at room temperature, powdered, and extracted following published procedure [14, 15]. Air-dried, coarsely ground plant samples (500 grams) were percolated four times with ethanol at room temperature, and the combined extracts were filtered and concentrated under reduced pressure in a thin film evaporator at 50 ±  °C (removing the solvent under pressure). Finally, the extract was completely dried under vacuum in the desiccators, which contain anhydrous calcium chloride. While in case of aqueous extracts, dried and powdered plant samples were soaked in sterile distilled water with constant stirring from 3 to 4 times till exhaustion of plant materials. The suspensions were further filtered through Whatman (No. 1) filter paper. Then, the filtrates were concentrated in vacuo using a rotary evaporator at 70°C then completely dried under vacuum in the desiccators.

### 2.2. Laboratory Animals Used

Guinea pigs 1-2 month, old were obtained from Helwan Farm of Laboratory Animals. (Ministry of Public Health). Animals were acclimatized to the animal house conditions (12 : 12 h light and dark cycle) for a week in galvanized zinc plated cages under strict hygienic conditions. The daily requirement of ascorbic acid (vitamin C) (50 mg/liter of drinking water) was supplied all over the experiment according to [16]. 

### 2.3. Preparation of the Inoculum

 The test organism (*E. coli*) was obtained from culture, from the Animal Health Research Institute, Giza. Serial dilution was prepared from the stock culture, and 1 mL from each dilution was IP administered into the Guinea pigs and watched for symptoms of infection. The dilution that established infection in the Guinea pigs which showed the symptoms and not high enough to cause rapid mortality was used as infectivity dosage for the Guinea pigs (1-2 × 10^8^) throughout the experiment.

### 2.4. Determination of Antibacterial Activity of the Extract

The antibacterial activity of the extracts (from leaves and root) by different solvent was determined by using disc diffusion method following published procedure. The test organisms were added separately to nutrient agar [17]. Sterile paper disc previously soaked in measured quantity of the sample (50 mg/mL and 100 mg/mL) was placed on plate containing solid bacterial medium. The plates were incubated aerobically at 37°C for 24 hrs and then examined for zones of inhibition with a ruler compared by +ve control (antibiotic disc) and −ve control (disc containing only physiological saline).

### 2.5. Infection and Treatment

The experiment was conducted with 50 1-2-month-old Guinea pigs. The animals were divided into 5 equal groups, 10 each. Control (Gp1) did receive neither viable bacteria nor treatment. On the other hand, each animal from the other groups (Gp 2–5) was challenged with (1-2 × 10^8^) viable *E. coli* in 200 *μ*L normal saline (0.9%) through IP route. GP2 infected group was treated with 200 *μ*L saline IP and kept as positive control group. (Gp 3-4) are infected and treated with *Withania somnifera* (ethanol root extract) with doses 50 and 100 mg/Kg BW, respectively. Gp5 is infected and treated group with cephalosporin antibiotic (cefoperazone) at dose 100 mg/Kg BW. The drug and medicinal plants doses change according to lab animals' body weight, which was calculated according to [18]. The treatment by drug or the extracted medicinal plant was started 72 h after infection for 7 successive days. Serum and whole blood sample were collected from all groups 14 days after treatment. 

### 2.6. Assessment of Bacterial Infection in the Vital Organs

To assess the efficacy of *Withania somnifera* plant extract treatment on the establishment of infection, five animals from each group were sacrificed on day 3 after infection and after end of the experiment, the different organs were aseptically removed and homogenized thoroughly in 5 mL sterile PBS, and then reisolation of the organism on solid media to observe the number of viable bacteria was done [7]. 

### 2.7. Haematological Test

Erythrogram (RBCs) and white blood cell count (WBC), calculation of blood indicesaccording to [19], and differential leukocytic count [20]. 

### 2.8. Serum Biochemical Analysis

Prepared frozen samples were used and analyzed for some serum analysis including ALT, AST, AP, urea, creatinine, glucose, cholesterol, total bilirubin, total protein, albumin, and globulin, determined with semiautomatic spectrophotometer (BM-Germany 5010) using commercial test kits (Randox Co., UK) according to enclosed pamphlet. 

### 2.9. Immunological Studies

Tumor necrosis factor-*α* (TNF-*α*) was assayed by Enzyme Amplified Sensitivity Immunoassay (EASIA) performed on microplate (Bio-Source, Co., Belgium). The assay uses monoclonal anti-bodies (MAbs) directed against distinct epitopes of TNF-*α* according to [21].

### 2.10. Statistical Analysis

Serum biochemical, hematological, and immunological parameters were analyzed by one way analysis of variance (ANOVA) using SPSS 20 for Windows. The mean and standard error were calculated for each variable. Pearson omnibus normality test was used to assess the normality of data distribution. Data were normally distributed; therefore, post hoc LSD multiple comparison was used to assess statistical differences among different groups. For all statistical examinations, results were considered significant at *P* < 0.05.

## 3. Results and Discussion

Our result (Table 1) demonstrates the potential efficacy of both leaves and root extracts (alcoholic and aqueous solvent) of *Withania somnifera* plant as remarkable antibacterial activity in vitro. Our result agrees with [6, 7], but the result of the study in vitro revealed that not all parts and solvents used give the same activity against the bacteria. This suggests that the antibacterial compound of* Withania somnifera* is polar in nature or at least exists either in the form of salt or glycoside under physical condition [22, 23].

Administration of *Withania somnifera* in our work in moderate doses did not lead to unfavorable hematological changes (Table 2). and The hemolytic effects of aqueous leaf extracts of *Withania somnifera* were demonstrated by incubating human erythrocytes with varying amount of crude extract (0–2 mg/mL) compared with chloramphenicol antibiotics, and the data show no lysis effect of the plant extract [7, 24]. In the same line, *W. somnifera* did not show any direct/indirect hemolytic activity against human and mice erythrocytes [9, 25]. 

Slight regenerative anemia appears then 14 days after treatment, in cefoperazone treated group (Table 2), and this result may be an evidence on the side effect of cefoperazone antibiotic which causes hemolysis of the RBCs and gastrointestinal hemorrhage [26] also it was summarized that several second and third generation cephalosporins have been associated with hypoprothrombinemia and bleeding [27]. 

We found that the erythron mass of the infected untreated group with *E. coli* revealed increase in the total RBCs, Hb concentration, and PCV%; this may be an indication of occurrence of polycythemia beside the significant increase of albumin and urea which confirms the dehydration occurrence [28]. 

In spite that *Withania somnifera* extract potential increase the total WBC count (Table 3) and increase the bone marrow cellularity of animals. Moreover, *Withania somnifera *root when administered orally at 25, 50, 100, and 200 mg/kg doses for 14 consecutive days showed a marked increase in the cell number of polymorphonuclear leucocytes cells [29, 30]. The results in this experiment revealed insignificant changes in TLC count and heterophils count in *Withania somnifera* extract treated group compared with the control but significantly decreased compared with infected untreated one, and this may be an indication on the antibacterial effect of this extract and it shows evidence that the *E. coli* was prevented from establishing infection. Christina et al. [31] recorded correction in the hematological parameter as significant decrease in WBCs while RBCs count was elevated in the cancer mice treated by root extract *Withania somnifera* and normal control group. These observations are suggestive of the protective effect of *Withania somnifera*.

It was cleared a slight significant leucopenia than control in drug treated group, but the decrease in heterophils count not significantly with the control (Table 3). It was reported that this drug and other beta-lactam antibiotics caused reversible neutropenia and transient eosinophilia has occurred. Also attributed to the mechanism of action of the drug, at which it was observed that the drug bind to both to neutrophils and bacteria in an individual manner and modify the binding and function of opsonins [32]. Increase in the TLC, heterophils count, and monocytes in infected untreated group is a signal that an infection has been established [33].

In *Withania somnifera* extract treated groups, there is a significant decrease in ALT and AST levels (Table 4) compared to infected group, and this may agree with the result assayed the hepatoprotective effect of this extract. Udayakumar et al. [34] reported that oral administration of *W. somnifera *extract for eight days to experimentally diabetic rats returns the liver transaminase enzymes to their normal level. Also Bhattacharya et al. [35], reported the hepatoprotective action of *W. somnifera* against iron-induced oxidative stress in rats after 10 days of oral administration of these active principles, in graded doses (10, 20, and 50 mg/kg).

The ALT and AST significantly increased in infected nontreated groups compared to other groups (Table 4), and this is indicative about the septicemic effect of bacteria; this result agrees with [36] who found that hepatic infection with was induced in the animals injected *E. coli* into the common bile duct.

No significance changed in biochemical parameters, total bilirubin, total protein, albumin, globulin, cholesterol, urea and creatinine (Tables 4 and 5) in *Withania somnifera* treated groups compared to control group. *Withania somnifera* is without having any serious toxicity or side effects known and thus can be safely used in humans for acute and chronic treatment regime [37]. Total plasma proteins were insignificant differ in *E. coli* and *Withania somnifera* in compare with control one (Table 3). *E. coli* infection caused hypoalbuminemia due to fall in the levels of albumin mRNA in response to infection parallel to a decrease in intrahepatic albumin synthesis due to liver damage. Also, infection can lead to increased catabolic rate and/or redistribution of albumin from plasma to interstitial compartment [38]. 

In the present study, there was a significant increase in blood urea level in comparison to control group (Table 5). In addition, transient elevations of the BUN and serum creatinine have been noted in patients during treatment with cefoperazone in dose from 43 to 75 mg/kg body weight, and also cefoperazone showed a low degree of nephrotoxicity. However, there was a decrease in glomerular filtration rate, which was seen in two patients treated for 3 weeks with a relatively high dose [39]. 

Serum TNF-*α* was highly significantly increased in *E. coli* infected group 12.33 ± 3.17 (pg/mL) compared to control, *Withania somnifera*, root ethanol extract at doses of 50 and 100 mg/kg Bw and cefoperazone treated group (57.33 ± 7.85, 14.66 ± 1.76, 16.06 ± 1.15, 9.50 ± 0.75), respectively. TNF-*α* is amplified, and coordinate proinflammatory sign also resulting in the synchronized expression of effectors molecules that mediate diverse aspects of innate immunity. TNF is capable of eliciting expression of chemokines and adhesion molecules and thus may be critical to the recruitment of neutrophils from the blood and has severe damage to the target organs [40, 41]. The transcription factor NF-*κ*B regulates the expression of cytokines, chemokines, adhesion factors, and inducible proinflammatory receptors. The abnormal activation of NF-*κ*B has been established for a series of inflammatory diseases and cancer. *Withania somnifera *is a medicinal plant that is widely used for the treatment of various inflammatory disorders. The leave extract of *Withania somnifera* potently inhibits NF-*κ*B activation by preventing the tumor necrosis factor. Results indicate that pure *W somnifera *extracts can be considered as a novel class of NF-*κ*B inhibitors, which hold promise as novel anti-inflammatory agents for treatment of various inflammatory disorders [42]. The effects of *Withania somnifera* on cytokine by IP administration of 20 mg of methanol root extract for 5 days and 10 days significantly increase in both IL-2 and IFN-*γ* while significant decrease in TNF-*α* was observed [43]. TNF-*α* was significantly increased in *E. coli* infected group comparied with control one. This result goes with [44], who recorded that the most marked acute-phase reactions in responses to *E. coli* are TNF-*α* responses in blood plasma. This result is also in accordance with [45], who reported IV administration of lethal dose of *E. coli* leading to increase of TNF-*α* levels.

## 4. Conclusion

From the previous result, we can conclude that *Withania somnifera* extract is potent antibacterial compound by in vitro methods and also as therapeutic in Guinea pig model of *Escherichia coli* infection. These appear in the correction of immunological, hematological, and biochemical alteration caused as response of infection. 

## Figures and Tables

**Table 1:** The antibacterial activity of (leaves and root) of *W.* *somnifera *against *E.* *coli* was determined by disc diffusion method.

	Zone of inhibition (in mm)				
Extract	Leaves extract			Root extract	
	50 mg/mL		100 mg/mL	50 mg/mL	100 mg/mL
	1	4 mm	6 mm	5 mm	11 mm
Aqueous	2	5 mm	5 mm	6 mm	10 mm
	3	5 mm	7 mm	5 mm	10 mm
	4	4 mm	5 mm	5 mm	9 mm

	1	5 mm	8 mm	8 mm	13 mm
Ethanol	2	6 mm	7 mm	6 mm	12 mm
	3	5 mm	8 mm	6 mm	12 mm
	4	6 mm	6 mm	8 mm	11 mm

**Table 2:** Erythrogram (mean ± S.E) at 14th day after treatment with cefoperazone and *Withania somnifera* extracts in Guinea pig experimental infected with *E.* *coli*.

Groups	RBC 10^6^/*μ*L	Hb Gm/dL	PCV %	MCV fl	MCH pg	MCHC %
I	4.71 ± 0.13^b^	13.36 ± 0.25^b^	37.50 ± 0.64^b^	79.58 ± 1.37^b^	28.34 ± 0.28^b^	35.62 ± 0.33^b^
II	5.26 ± 0.14^a^	16.99 ± 0.41^a^	41.50 ± 0.64^a^	78.97 ± 2.17^b^	32.13 ± 0.87^a^	40.94 ± 0.40^a^
III	4.62 ± 0.14^b^	12.84 ± 0.41^bc^	37.50 ± 0.64^b^	81.24 ± 2.26^b^	27.77 ± 0.48^b^	34.28 ± 1.27^bc^
IV	4.55 ± 0.13^b^	13.29 ± 0.43^b^	37.75 ± 0.62^b^	82.95 ± 1.7^ab^	29.16 ± 0.56^ab^	35.17 ± 0.65^ b^
V	4.11 ± 0.18^c^	11.79 ± 0.60^c^	36.50 ± 0.6^b^	88.99 ± 2.70^a^	28.62 ± 0.52^b^	32.26 ± 1.34^c^

The same column not followed by the same letter differs significantly (*P* < 0.05).

**Table 3:** Leukogram (mean ± S.E) at 14th day after treatment with cefoperazone and *Withania somnifera* extracts in Guinea pig experimental infected with *E.* *coli*.

Groups	TLC 10^3^/*μ*L	Heterophil 10^3^/*μ*L	Esinophil 10^3^/*μ*L	Basophil 10^3^/*μ*L	Lymphocyte 10^3^/*μ*L	Monocyte 10^3^/*μ*L
I	8.31 ± 0.37^b^	2.86 ± 0.23^bc^	0.46 ± 0.13^a^	0.02 ± 0.02^a^	4.41 ± 0.54^a^	0.49 ± 0.10^cb^
II	11.94 ± 0.78^a^	6.13 ± 0.38^a^	0.33 ± 0.11^a^	0.11 ± 0.02^a^	4.29 ± 0.63^a^	1.05 ± 0.11^a^
III	6.65 ± 0.42^cb^	2.44 ± 0.28^c^	0.34 ± 0.16^a^	0.02 ± 0.02^a^	3.51 ± 0.33^ab^	0.42 ± 0.10^cb^
IV	7.89 ± 0.21^b^	3.65 ± 0.53^bc^	0.58 ± 0.14^a^	0.02 ± 0.02^a^	3.21 ± 0.21^ab^	0.35 ± 0.10^cb^
V	6.18 ± 0.47^c^	2.34 ± 0.28^c^	0.44 ± 0.02^a^	0.15 ± 0.02^a^	2.96 ± 0.45^b^	0.28 ± 0.02^c^

The same column not followed by the same letter differs significantly (*P* < 0.05).

**Table 4:** Some selective biochemical parameters (mean ± S.E), in Guinea pig experimental infected with *E.* *coli. *treated *Withania somnifera* extracts.

Groups	ALT U/L	AST U/L	AP U/L	T. Bili. mg/dL	Chol. mg/dL	Glu. mg/dL
I	14.25 ± 1.7^c^	31.5 ± 1.92^c^	19.25 ± 1.49^a^	0.39 ± 0.013^a^	33.75 ± 3.47^b^	106.25 ± 6.25^a^
II	32.00 ± 1.58^a^	50.50 ± 2.39^a^	19.75 ± 2.49^a^	0.44 ± 0.05^a^	43.75 ± 2.81^a^	100.75 ± 5.4^a^
III	25.50 ± 2.53^b^	35.75 ± 1.43^cb^	16.50 ± 1.32^a^	0.39 ± 0.011^a^	28.50 ± 1.50^b^	75.50 ± 6.06^b^
IV	24.50 ± 3.27^b^	37.50 ± 2.02^b^	19.00 ± 1.87^a^	0.36 ± 0.033^a^	26.75 ± 4.2^b^	111.20 ± 4.49^a^
V	24.75 ± 2.32^b^	41.00 ± 1.87^b^	16.50 ± 1.70^a^	0.35 ± 0.02^a^	30.50 ± 2.32^b^	98.25 ± 4.64^a^

The same column not followed by the same letter differs significantly (*P* < 0.05).

AP: alkaline phosphatase; T. Bili.: total bilirubin; Chol.: cholesterol; Glu.: glucose.

**Table 5:** Some selective biochemical parameters (mean ± S.E), in Guinea pig experimental infected with *E.* *coli. *treated *Withania somnifera* extracts.

Groups	T.P. gm/dL	Alb. gm/dL	Glob. gm/dL	Urea mg/dL	Creat. mg/d
I	4.57 ± 2.59^a^	2.75 ± 0.29^b^	1.80 ± 0.29^a^	39.50 ± 2.25^c^	0.81 ± 0.04^b^
II	5.95 ± 0.38^a^	3.92 ± 0.22^a^	2.02 ± 0.26^a^	57.50 ± 2.21^a^	1.01 ± 0.08^a^
III	4.57 ± 0.38^a^	2.97 ± 0.26^b^	1.60 ± 0.29^a^	37.00 ± 2.27^c^	0.69 ± 0.031^b^
IV	4.87 ± 0.49^a^	3.1 ± 0.32^ab^	1.77 ± 0.42^a^	37.75 ± 2.01^c^	0.88 ± 0.14^ab^
V	4.77 ± 0.82^a^	2.77 ± 0.37^b^	2.00 ± 0.56^a^	48.75 ± 1.10^b^	0.86 ± 0.03^ab^

The same column not followed by the same letter differs significantly (*P* < 0.05).

T.P.: total protein; Alb.: albumin; Glob.: globulin; Creat.: creatinine.

## References

[1] Modak M, Dixit P, Londhe J, Ghaskadbi S, Devasagayam TPA (2007). Indian herbs and herbal drugs used for the treatment of diabetes. *Journal of Clinical Biochemistry and Nutrition*.

[2] Veerappan A, Miyazaki S, Kadarkaraisamy M, Ranganathan D (2007). Acute and subacute toxicity studies of *Aegle marmelos* Corr., an Indian medicinal plant. *Phytomedicine*.

[3] Mukhtar M, Arshad M, Ahmad M, Pomerantz RJ, Wigdahl B, Parveen Z (2008). Antiviral potentials of medicinal plants. *Virus Research*.

[4] Girdhari L, Rana A (2007). *Withania somnifera* (Ashwagandha): a review. *Pharmacognosy Reviews*.

[5] Fawzy TKH (1983). *Medicinal Plants in Lybya*.

[6] Arora S, Dhillon S, Rani G, Nagpal A (2004). The in vitro antibacterial/synergistic activities of *Withania somnifera* extracts. *Fitoterapia*.

[7] Owais M, Sharad KS, Shehbaz A, Saleemuddin M (2005). Antibacterial efficacy of *Withania somnifera* (Ashwagandha) an indigenous medicinal plant against experimental murine salmonellosis. *Phytomedicine*.

[8] Teixeira ST, Valadares MC, Gonçalves SA, de Melo A, Queiroz MLS (2006). Prophylactic administration of *Withania somnifera* extract increases host resistance in *Listeria monocytogenes* infected mice. *International Immunopharmacology*.

[9] Ziauddin M, Phansalkar N, Patki P, Diwanay S, Patwardhan B (1996). Studies on the immunomodulatory effects of Ashwagandha. *Journal of Ethnopharmacology*.

[10] Dhuley JN (1997). Effect of some Indian herbs on macrophage functions in ochratoxin A treated mice. *Journal of Ethnopharmacology*.

[11] Davis L, Kuttan G (2000). Immunomodulatory activity of *Withania somnifera*. *Journal of Ethnopharmacology*.

[12] Mehta M, Dutta P, Gupta V (2005). Antimicrobial susceptibility pattern of blood isolates from a teaching hospital in North India. *Japanese Journal of Infectious Diseases*.

[13] Shimizu K (1980). Cefoperazone: absorption, excretion, distribution, and metabolism. *Clinical Therapeutics*.

[14] Malik F, Singh J, Khajuria A (2007). A standardized root extract of *Withania somnifera* and its major constituent withanolide-A elicit humoral and cell-mediated immune responses by up regulation of Th1-dominant polarization in BALB/c mice. *Life Sciences*.

[15] Sreemantula S, Nammi S, Kolanukonda R, Koppula S, Boini KM (2005). Adaptogenic and nootropic activities of aqueous extract of *Vitis vinifera* (grape seed): an experimental study in rat model. *BMC Complementary and Alternative Medicine*.

[16] Sarah W, Maggie L (2003). *Handbook of Laboratory Animal Management and Welfare*.

[17] Akinyemi KO, Mendie UE, Smith ST, Oyefolu AO, Coker AO (2005). Screening of some medicinal plants used in south-west Nigerian traditional medicine for anti-salmonella typhi activity. *Journal of Herbal Pharmacotherapy*.

[18] Paget GE, Barnes JM (1964). *Evaluation of Drug Activities Pharmacometrics*.

[19] Feldman BF, Zinkl JG, Jain VC (2000). Laboratory techniques for avian hematology. *Schalm's Veterinary Hematology*.

[20] Coles EH (1986). *Veterinary Clinical Pathology*.

[21] Aukrust P, Liabakk NB, Muller F, Lien E, Espevik T, Froland SS (1994). Serum level of tumor necrosis factor-*α* and soluble TNF receptors in human immunologic virus type 1 infection correlation to clinical immunologic and virologic parameters. *Journal of Infectious Diseases*.

[22] Mazzanti G, Mascellino MT, Battinelli L, Coluccia D, Manganaro M, Saso L (2000). Antimicrobial investigation of semipurified fractions of *Ginkgo biloba* leaves. *Journal of Ethnopharmacology*.

[23] Elsakka M, Grigorescu E, Stanescu U, Stanescu U, Dorneanu V (1990). New data referring to chemistry of *Withania somnifera* species. *Revista Medico-Chirurgicala a Societatii de Medici si Naturalisti din Iasi*.

[24] He XG, Mocek U, Floss HG (1994). An antifungal compound from *Solanum nigrescens*. *Journal of Ethnopharmacology*.

[25] Girish KS, Machiah KD, Ushanandini S (2006). Antimicrobial properties of a non-toxic glycoprotein (WSG) from *Withania somnifera* (Ashwagandha). *Journal of Basic Microbiology*.

[26] Kumar AV, Rao NR (1999). Cefoperazone induced gastrointestinal haemorrhage. *The Journal of the Association of Physicians of India*.

[27] Schentag JJ, Welage LS, Grasela TH, Adelman MH (1987). Determinants of antibiotic-associated hypoprothrombinemia. *Pharmacotherapy*.

[28] Kaneko JJ, John WH, Micheal LLB (1997). *Clinical Biochemistry of Domestic Animals*.

[29] Kuttan G (1996). Use of *Withania somnifera* Dunal as an adjuvant during radiation therapy. *Indian Journal of Experimental Biology*.

[30] Khan B, Ahmad SF, Bani S (2006). Augmentation and proliferation of T lymphocytes and Th-1 cytokines by *Withania somnifera* in stressed mice. *International Immunopharmacology*.

[31] Christina AJM, Gladwin JD, Packialakshmi M (2004). Anticarcinogenic activity of *Withania somnifera* Dunal against Dalton’s Ascitic Lymphoma. *Journal of Ethnopharmacology*.

[32] Strom BL, Schinnar R, Gibson GA, Brennan PJ, Jesse A (1999). Risk of bleeding and hypoprothrombinaemia associated with NMIT side chain antibiotics: using cefoperazone as a test case. *Pharmacoepidemiology and Drug Safety*.

[33] Ogundare AO, Onifade AK (2009). The antimicrobial activity of Morinda lucida leaf extract on *Escherichia coli*. *Journal of Medicinal Plant Research*.

[34] Udayakumar R, Kasthurirengan S, Mariashibu TS (2009). Hypoglycaemic and hypolipidaemic effects of *Withania somnifera* root and leaf extracts on alloxan-induced diabetic rats. *International Journal of Molecular Sciences*.

[35] Bhattacharya A, Ramanathan M, Ghosal S, Bhattacharya SK (2000). Effect of *Withania somnifera* Glycowithanolides on iron-induced hepatotoxicity in rats. *Phytotherapy Research*.

[36] Nagano I, Kato S, Nimura Y, Wakabayashi T (1994). Hepatic mitochondrial changes in experimental obstructive jaundice complicated by biliary infection. *Hepato-Gastroenterology*.

[37] Kulkarni SK, Dhir A (2008). *Withania somnifera*: an Indian ginseng. *Progress in Neuro-Psychopharmacology and Biological Psychiatry*.

[38] Benoit R, Isabelle P, Fabienne B, Christiane O (2000). Increased albumin plasma efflux contributes to hypoalbuminemia only during early phase of sepsis in rats. *American Journal of Physiology*.

[39] Trollfors B, Ahlmen J, Alestig K (1982). Renal function during cefoperazone treatment. *Journal of Antimicrobial Chemotherapy*.

[40] Isogai E, Isogai H, Kimura K (1998). Role of tumor necrosis factor alpha in gnotobiotic mice infected with an *Escherichia coli* O157 : H7 Strain. *Infection and Immunity*.

[41] Mizgerd JP, Spieker MR, Doerschuk CM (2001). Early response cytokines and innate immunity: essential roles for TNF receptor 1 and type I IL-1 receptor during *Escherichia coli* pneumonia in mice. *Journal of Immunology*.

[42] Kaileh M, vanden Berghe W, Heyerick A (2007). Withaferin A strongly elicits I*κ*B kinase *β* hyperphosphorylation concomitant with potent inhibition of its kinase activity. *The Journal of Biological Chemistry*.

[43] Davis L, Kuttan G (1999). Effect of *Withania somnifera* on cytokine production in normal and cyclophosphamide treated mice. *Immunopharmacology and Immunotoxicology*.

[44] Theodore JS (2000). Anti-inflammatory cytokines and cytokine antagonists. *Current Pharmaceutical Design*.

[45] Zimecki M, Artym J, Chodaczek G, Kocieba M, Kruzel ML (2004). Protective effects of lactoferrin in *Escherichia coli* -induced bacteremia in mice: relationship to reduced serum TNF alpha level and increased turnover of neutrophils. *Inflammation Research*.

